# Coreceptor usage of plasma and cerebrospinal fluid-derived HIV-1 subtype C variants

**DOI:** 10.1186/s12985-026-03150-0

**Published:** 2026-04-04

**Authors:** Suhina Sirkisson, Nelisiwe Zikhali, Christina Chang, Thumbi Ndung’u, Katlego Sojane, Bongiwe Ndlovu

**Affiliations:** 1 HIV Pathogenesis Programme, The Doris Duke Medical Research Institute, University of KwaZulu-Natal, Durban, South Africa; 2 Department of Infectious Diseases, Alfred Hospital, Melbourne, Australia; 3 Department of Infectious Diseases, School of Translational Medicine, Monash University, Melbourne, Australia; 4 Africa Health Research Institute, Durban, South Africa; 5 Ragon Institute of Massachusetts General Hospital, Massachusetts Institute of TechnologyandHarvard University, Cambridge, MA USA; 6 Division of Infection and Immunity, University College London, London, UK

**Keywords:** HIV-1C, Envelope, Coreceptor usage, CNS and peripheral blood

## Abstract

**Background:**

HIV-1 subtype C (HIV-1C) may use multiple coreceptors to enter cells. However, the differences in coreceptor usage between HIV-1C Env variants from peripheral blood and those from the central nervous system (CNS) are still not completely understood. This study characterised the coreceptor usage of viral populations that circulate in the central nervous system (CNS) and plasma compartments to enhance understanding of HIV-1C pathogenesis and inform the appropriate use of entry inhibitors.

**Methods:**

We characterised the in vitro coreceptor usage of HIV-1C envelope protein (Env) variants circulating in peripheral blood and the cerebrospinal fluid (CSF) of twelve participants living with HIV and cryptococcal meningitis coinfection. We evaluated the coreceptor usage using Geno2pheno, Phenoseq and Web-PSSM coreceptor prediction tools, and confirmed coreceptor usage in U87 cell lines. We also analysed HIV-1 Env V3 loop characteristics for associations with CCR5 or CXCR4 usage.

**Results:**

Our findings indicated that CSF-derived clones primarily used CCR5, while plasma-derived clones varied usage between CCR5, CXCR4 and CCR3 in vitro. HIV-1 Envs with a long V3 loop (37 amino acids), absence of potential N-linked glycosylation sites (PNGS), and a high net charge (> 5 +) were associated with CXCR4 usage. Whereas shorter or standard-length V3 loops (34–35 amino acids), presence of PNGS, lower net charge (range + 2 to + 5) and a GPGQ crown motif were associated with CCR5 usage.

**Conclusion:**

Our findings reveal compartment-specific heterogeneity in HIV-1 subtype C coreceptor usage during advanced infection. CSF-derived variants were primarily limited to CCR5 usage, while plasma-derived variants exhibited broader usage of CXCR4 and alternative coreceptors. Phenotypic validation, together with distinct V3 loop signatures, provides evidence of viral adaptation to divergent tissue environments and highlights limitations of genotypic tropism prediction alone. These findings advance the understanding of HIV-1C pathogenesis and have implications for evaluating coreceptor usage in the context of entry inhibitor–based interventions particularly in regions where HIV-1C is endemic and diagnostic resources are limited.

**Supplementary Information:**

The online version contains supplementary material available at 10.1186/s12985-026-03150-0.

## Background/Introduction:

HIV-1 remains a major public health problem globally. In 2024, there were approximately 39,9 million people living with HIV (PLWH), and 630,000 people died due to HIV-related illnesses [1]. Current biomedical prevention and treatment strategies include the administration of antiretroviral therapy (ART), pre-exposure prophylaxis (PrEP), medical male circumcision and treatment of sexually transmitted infections (STIs) [2]. However, these interventions are sub-optimal and are associated with various challenges, including inadequate access to healthcare systems, lack of awareness and uptake of PrEP, non-adherence to ART and drug toxicity [1–3]. This highlights the need for more effective prevention and treatment strategies.

Several antiretroviral drugs including fostemsavir (anti-gp120), ibalizumab (anti-CD4 monoclonal antibody) and maraviroc (anti-CCR5) were designed to prevent HIV-1 entry and reduce viral replication in participants who have drug resistance to most currently available drugs [4]. In particular, maraviroc (MVC), competitively binds the gp120 V3 loop and prevents interaction with CCR5 coreceptors [5–7]. As such, it was used in the treatment of patients with resistance to other ART [8–10]. However, MVC has seen limited use in sub-Saharan Africa due to its relatively high cost and associated diagnostic costs for viral coreceptor usage determination, which are largely unaffordable in low- or middle-income countries. These challenges underscore the need for new drug discovery or repurposing for PLWH and the development of new tools for determining the coreceptor usage.

HIV-1 clade C (HIV-1C) predominantly circulates in Southern Africa, Ethiopia, and India, and accounts for 47% of global infections [11]. In peripheral blood, HIV-1C primarily infects CD4 + T lymphocytes and myeloid cells using either the CCR5 or CXCR4 coreceptors [12–15]. CCR5 is expressed on monocytes, macrophages, T lymphocytes, natural killer cells, and dendritic cells [12–14, 16]. In contrast, CXCR4 is expressed on a broader range of CD4 + T-cell populations, including thymocytes, hematopoietic progenitor cells, stromal fibroblasts, endothelial cells, and macrophages [12–15]. HIV-1C predominantly uses CCR5 (R5 viruses) to establish an infection [17, 18]. On the other hand, HIV-1C variants capable of using CXCR4 alone (X4 viruses) or in combination with CCR5 (R5X4 viruses) were reported to arise mainly during chronic infection [19, 20]. Notably, studies have suggested that the general prevalence of R5X4/X4 viruses is increasing over time [21–24], even in acute infections [14, 25, 26]. A recent study reported a predicted prevalence of ~ 9% of early-chronic infection patients with R5X4/X4 viruses across Africa, with a three–fivefold increase in prevalence specifically observed during chronic infection [27]. Interestingly, South Africa displayed the highest predicted prevalence of R5X4/X4 viruses compared to other African countries [27].

CXCR4 usage is associated with accelerated loss of CD4 + T-cells and increased progression to AIDS in various HIV subtypes [15, 28–31]. Furthermore, it was shown that the switch from CCR5 to CXCR4 usage may occur as a result of resistance mutations to protease inhibitors and V3-specific broadly neutralising antibodies (bnAbs) [32]. Notably, the timing and determinants of coreceptor usage, particularly CXCR4 usage, are not completely clear. Characterising HIV-1C coreceptor usage may therefore assist in clarifying the mechanisms, timing and reasons for HIV-1C modifying its coreceptor usage. Beyond primary CCR5 and CXCR4 coreceptors, HIV-1 has demonstrated the ability to use alternative coreceptors, including CCR3 (R3 viruses) [8, 33–42]. Notably, an expanded coreceptor usage profile for subtype C has been previously observed and has generally been reported in individuals with chronic or end-stage disease [14, 43–46]. Understanding the relevance of expanded coreceptor usage profile is critical to assess HIV-1C pathogenesis and the impact it may have on the efficacy of potential treatment.

HIV-1 coreceptor usage can vary between anatomical compartments [47–51]. The central nervous system (CNS) is an anatomical compartment with unique selection pressures, including target cell types, immune microenvironment and drug penetration compared to peripheral blood [52, 53]. This infected cell profile shifts in the CNS, where the virus primarily infects perivascular macrophages, microglia, and astrocytes, which notably mostly express CCR5 [54, 55]. Unlike short-lived CD4 + T-cells, these CNS-resident cells can persist for weeks to months following infection, serving as long-lived HIV reservoirs [56].

Previous studies reported genotypic differences in the HIV-1 *env* V3-loop and the CD4-binding site between CNS and plasma-derived sequences [12, 57, 58]. Additionally, studies have also observed different viral loads and white blood cell counts between the CNS and the plasma compartments [59, 60]. While concordant coreceptor usage between the compartments is generally observed, compartmentalisation may arise [47–51]. Coreceptor usage of HIV-1 in the CSF is predominantly CCR5, but R5X4 variants have also been observed [51]. Additionally, a previous study reported expanded coreceptor usage, including CCR3, within the CNS [51]. Compared to the CSF, plasma samples display an expanded coreceptor usage profile (beyond CCR3, CCR5 and CXCR4) and more frequent occurrence of X4 viruses [48]. However, the majority of coreceptor usage studies used coreceptor prediction algorithms and few studies confirmed coreceptor usage using tissue culture-based methods. Some studies have shown that prediction tools are not always accurate and may need further development. While CCR5-tropic HIV-1C remains predominant, the increasing occurrence of CXCR4- and alternate coreceptor-using viral variants in advanced infection highlights the clinical importance of coreceptor switching especially in the context of pathogenesis, disease progression and treatment efficacy. Accurately characterising HIV-1C coreceptor usage, in vitro, is essential to improve our understanding of the context and timing of coreceptor switching and to effectively identify individuals who are at risk of rapid disease progression or those eligible for certain entry inhibitor therapies such as maraviroc, and therefore improving the design and selection of HIV prevention or treatment strategies. The purpose of this study was to characterise the in vitro coreceptor usage of the CSF- and plasma-derived HIV-1C Env using a phenotypic assay. Furthermore, we aimed to identify genotypic properties associated with coreceptor usage profiles.

## Methods

### Study Participants

Twelve participants (five women and seven men) in this sub-study were selected from a larger longitudinal clinical study of people living with HIV-1 (PLWH) and had cryptococcosis-associated immune reconstitution inflammatory syndrome (C-IRIS). The study participants were recruited from 2009 to 2011 in Durban, South Africa, were ART-naïve, between 23 and 45 years of age who had experienced their first episode of cryptococcal meningitis (CM) [61]. The participants had either a positive cerebrospinal fluid (CSF) cryptococcal antigen (CrAg) or India ink test. They received intravenous amphotericin for 14 days; this was followed by fluconazole as maintenance therapy and standard combined antiretroviral therapy (cART). Cerebrospinal fluid (CSF) and plasma samples were collected at baseline before cART initiation (Table 1). Written informed consent was obtained from the study participants or their next of kin. Ethics approval for this study was received from the Biomedical Research and Ethics Committee of the University of KwaZulu-Natal (BF053/09 and BREC/00001996/2020), Monash University (2,009,001,224) and the University of Western Australia (RA/4/1/2541).

**Table 1:** The demographic and clinical characteristics of the study participants

PID	Sex	Age	CD4⁺ T cell count	Plasma VL	CSF VL	CSF (WBC)
CM019	M	45	7	5.15	4.33	28
CM021	F	30	134	4.34	4.34	40
CM029	F	33	121	5.22	5.99	424
CM032	F	34	5	4.94	2.52	0
CM052	F	24	172	4.62	4.83	36
CM054	M	23	7	5.20	4.20	14
CM070	M	25	11	5.04	4.00	18
CM089	M	27	114	5.67	5.85	154
CM094	F	29	35	5.15	4.03	84
CM098	M	40	14	4.76	5.27	66
CM108	M	27	20	5.16	5.02	28
CM132	M	34	53	4.58	4.43	38
**Median:**	**-**	**29.50**	**27.50**	**5.10**	**4.39**	**37**

PID = patient identification number, CD4 + count = CD4 + T cell count measured as cells/μL, VL = HIV-1 RNA viral load (VL) measured as log10 copies/mL, and WBC = white blood cell count measured as cells/μL

### Viral load, CD4 T cell count and cryptococcal meningitis screening

We measured HIV-1 viral load (detection limit 34 copies/mL) in the CSF and plasma samples using the COBAS TaqMan HIV-1 kit according to the manufacturer’s instructions (Hoffman-La Roche, Basel, Switzerland). Total CSF white blood cells (WBC) and whole blood CD4^+^ T cell counts were measured using Tru-Count technology and analysed by four-colour flow cytometry (Becton Dickinson, New Jersey, USA). Gram staining and Cryptococcal antigen (CrAg) or Indian ink tests were used to confirm cryptococcal meningitis coinfection [61].

### RNA extractions, cDNA synthesis

RNA was extracted from plasma and CSF using the QIAamp Viral RNA Mini kit according to the manufacturer’s instructions (Qiagen, Dusseldorf, Germany). Complementary DNA (cDNA) was synthesised using SuperScript III Reverse Transcriptase (Invitrogen, Carlsbad, CA) and OFM19 primer (5’-GCACTCAAGGCAAGCTTTATTGAGGCTTA-3’; HXB2 positions 9,604–9,632) as described previously [62].

### Single genome amplification

Full-length HIV-1 *env* was amplified from cDNA using single-genome amplification (SGA) as described previously [24, 62]. Briefly, the synthesized cDNA was serially diluted, with at least 10 PCR reactions for each dilution. The endpoint dilution was defined as ≤ 30% positive amplification reactions, so that each positive amplification corresponded to a unique viral variant template [63]. Platinum Taq DNA Polymerase High Fidelity (Invitrogen, Carlsbad, CA) was used for both rounds of amplification as previously described [62, 64]. The initial PCR round was performed with the forward primer VIF1 (5’-GGGTTTATTACAGGGACAGCAGAG-3’; HXB2 positions 4,900–4,923), the reverse primer OFM19, and 1 μL of diluted cDNA. The second round of PCR was conducted using the forward primer ENVA (5’-GCTTAGGCATCTCCTATGGCAGGAAGAA-3’; HXB2 positions 5,945–5,982), reverse primer ENVN (5’-CTGCCAATCAGGGAAGTAGCCTTGTGT-3’; HXB2 positions 9,145–9,171) and 2 μL of first-round PCR product. Both rounds of PCR had the following thermal cycling conditions: 94 °C for 4 min; 35 cycles of 94 °C for 15 s, 55 °C for 30 s, 68 °C for 4 min; a final extension step of 68 °C step for 20 min followed by a hold at 4 °C. The second-round PCR products were analysed on a 1% agarose gel to identify positive PCR reactions.

### Bulk PCR

The 2.1 KpnI-to-BamHI *env* gene fragments were amplified from undiluted cDNA with a modified, nested bulk PCR and cloning protocol, as described previously [65–67]. The first-round PCR mixture included the forward VIF1 primer, reverse OFM19 primer, the Platinum Taq DNA Polymerase High Fidelity (Invitrogen, Carlsbad, CA), and 1 μL of undiluted cDNA. The second round PCR was performed using the Env-KpnI forward primer (5’-GTCTATTATGGGGTACCTGTGTGG-3’; HXB2 positions 6,336–6,359, 20 mM), the Env-BamHI reverse primer (5’-GCTAAGGATCCGTTCACTAATCGT-3’; HXB2 position 8,463–8,485, 20 mM), the Phusion High-Fidelity DNA polymerase (New England Biolabs, Ipswich, MA), and 5 µL of first round PCR product. The second-round PCR products were analysed by 1% agarose gel electrophoresis and any amplicons detected were then gel-purified (Invitrogen, Carlsbad, CA).

### Cloning

The 2.1 kb Env amplicons generated using the first-round SGA or bulk PCR amplification products, and the pSVIIIenv plasmid were digested separately with BamHI and ACC65I restriction enzymes (Thermo Fisher Scientific, Waltham, MA) and ligated together using the Rapid DNA Ligation Kit (Thermo Fisher Scientific, Waltham, MA), as previously described [65, 67, 68]. After ligation, the ligation mixture containing recombinant plasmids was used to transform XL10-Gold Ultracompetent Cells (Agilent Technologies, Santa Clara, CA). Subsequently, the transformation mixture was inoculated on ampicillin-agar plates (100 μg/mL ampicillin) according to the manufacturer’s instructions. PCR screening of the resulting colonies was performed with the Env-KpnI (20 μM) and Env-BamHI (20 μM) primers and SuperTherm Taq (Separation Scientific, Johannesburg, South Africa) according to the manufacturer’s instructions.

### Sanger sequencing and phylogenetic analyses

Full-length HIV-1 *env* was sequenced via Sanger sequencing using the ABI Prism Big Dye Terminator Version 3.1 sequencing kit according to the manufacturer’s instructions (Applied Biosystems, Foster City, CA). Eight HIV-1C *env* forward and reverse primers were used to prepare the sequencing reactions as previously described [69]. The sequences were resolved on the ABI 3130 XL genetic analyser, and the contigs were assembled and edited using Sequencher v4.8 (Genecodes, Ann Arbor, MI) as previously described [64]. Sanger sequencing was used to validate the insertion of *env* fragments into pSVIII vector, due to its high accuracy and precision. Phylogenetic analyses were performed using IQ-TREE available on the Los Alamos National Laboratory database (https://www.hiv.lanl.gov/content/sequence/IQTREE/iqtree.html). Briefly, the V1-V5 *env* sequences were aligned in MEGA X [70] and the phylogenetic relatedness of HIV-1 *env* was determined using the Maximum Likelihood method with 1,000 bootstrap replicates. The phylogenetic tree was further edited using Rainbow tree (https://www.hiv.lanl.gov/content/sequence/RAINBOWTREE/rainbowtree.html). Thereafter, we used the phylogenetic tree to evaluate the phylogenetic-relatedness of the sequences collected.

### Cell lines

The 293 T cells and TZM-bl cells were obtained from ATCC (American Type Culture Collection, Manassas, VA) and the NIH HIV Reagent Program (AIDS Reagent Program, Bethesda, MD, USA), respectively. The TZM-bl cells express CD4, CCR5 and CXCR4 and have a Tat-inducible luciferase reporter gene [71, 72]. Both the 293 T and TZM-bl cell lines were maintained in complete Dulbecco’s Modified Eagle Media (DMEM) (Life Technologies, Carlsbad, CA, USA) supplemented with 10% foetal bovine serum (Gibco BRL Life Technologies, Carlsbad, CA, USA) and 1% penicillin/streptomycin (Thermo Fisher Scientific, Johannesburg, South Africa) [DMEM ( +)]. U87-CD4 + cells expressing CD4 and either CCR3, CCR5, or CXCR4 coreceptors [34, 73, 74] were obtained from the NIH HIV Reagent Program (AIDS Reagent Program, Bethesda, MD, USA). Each U87-CD4 + cell line was maintained in DMEM, supplemented with 1 μg/mL of puromycin (Sigma-Aldrich, St. Louis, MO) and 300 μg/mL of G418 [DMEM (+ +)] (Thermo Fisher Scientific, Johannesburg, South Africa). The cell lines in this sub-study were all incubated at 37 °C and 5% CO_2_.

### Pseudovirus generation and functionality testing

Participant-derived HIV-1C pseudoviruses and HIV-1B reference pseudoviruses [YU2 (R5), NL4.3 (X4) and 89.6 (R3R5X4)] were generated in 293 T cells as previously described [75]. All of the single-round infection pseudoviruses were generated via co-transfection of recombinant pSVIIIenv, pCMVΔP1Δ and pHIV1LUC in 293 T cells using polyethyleneimine (PEI) transfection reagent (Sigma-Aldrich). The transfected cells were incubated for 48 h at 37 °C, and in 5% CO_2_ [66]. After incubation, the supernatants were harvested, passed through 0.45 μm filters and concentrated by centrifugation at 4,500 × g, for 6 min using Amicon Ultra-15 centrifugal filter units (Merck, Darmstadt, Germany). After concentrating the viral suspension, the supernatants were aliquoted and stored at -80 °C.

The infectivity of the luciferase-reporter HIV-1 pseudoviruses was tested in TZM-bl cells as described previously [75]. Five-fold serial dilutions (1:1, 1:5, 1:25, and 1:125) of pseudovirus and a negative control (culture medium only) were prepared, in triplicate, in 96-well tissue culture plates (Corning, New York, USA). Freshly trypsinized TZM-bl cells (1 X 10^4^ cells/well) in 100 μL DMEM ( +) containing 60 µg/mL of DEAE-dextran were added, and the plates were incubated for 48 h at 37 °C and 5% CO_2_. After incubation, the supernatant was removed and the cells were washed with phosphate-buffered saline (PBS) (Thermo Fisher Scientific, Waltham, MA) and lysed with the Cell Culture Lysis 5 × Reagent (Promega, Wisconsin, USA). Luciferase activity in cell lysates was measured using the Luciferase Assay System (Promega, Wisconsin, USA) and the Victor Nivo Multimode Microplate Reader (PerkinElmer, Massachusetts, USA) according to the manufacturer’s instructions. Pseudoviruses that generated relative light unit (RLU) values (arbitrary units) that were three-fold greater than the negative control were classified as infectious.

### In vitro* coreceptor usage of HIV-1C in U87-CD4* + *cells*

We determined the in vitro coreceptor usage of plasma and CSF-derived Envs in U87-CD4 + cells expressing CCR3, CCR5 or CXCR4. U87-CD4 + cells expressing CCR3, CCR5 or CXCR4 (1 X 10^4^/well) in 100 µL of DMEM (+ +) were seeded in 96-well plates and incubated for 12 h at 37 °C, and 5% CO_2_. Thereafter, 100 µL of undiluted HIV-1C pseudovirus was added to the cells and incubated for another 24 h at 37 °C, 5% CO_2_. The supernatant was replaced with fresh culture media and incubated for a further 48 h. At 72 h post-infection, the culture medium was discarded, the cells were washed with PBS, lysed using the Cell culture lysis 5 × reagent (Promega, Wisconsin, USA) and the pseudovirus infectivity was measured on the Victor Nivo Multimode plate reader. A total of eight technical repeats were conducted. Pseudoviruses that generated RLU values, which were five-fold greater than those of the mock infection control were classified as using the coreceptor expressed on the cell. HIV-1 subtype B pseudoviruses (89.6, NL4.3, and YU2) were included as reference controls.

### HIV-1 env sequence analyses

To identify amino acid signatures and genotypic features associated with coreceptor usage profiles, HIV-1C *env* sequences from twelve participants were aligned with HXB2 and translated using the Gene Cutter tool available on the Los Alamos National Laboratory database (https://www.hiv.lanl.gov/content/sequence/GENE_CUTTER/cutter.html). Amino acid sequences were analysed and associated with CCR5 and CXCR4 coreceptor usage as described previously [76, 77]. The crown motif, V3 loop length, overall net charge, and potential N-linked glycosylation sites (PNGS) in the V3 loop of each envelope were determined using the Variable Region Characteristics tool also available on the Los Alamos National Laboratory database (https://www.hiv.lanl.gov/content/sequence/VAR_REG_CHAR/index.html).

### Statistical analysis

A two-tailed unpaired nonparametric t-test was used to compare median viral loads between the plasma and the CSF. We used Spearman’s correlation test to assess the correlation between the CD4 + T-cell count and the white blood cell count in the CSF. A significant difference was defined as the p < 0.05. In vitro coreceptor usage results were analysed using Prism Version 9.4 (Graphpad, San Diego, CA), with the median and range of replicates calculated and reported.

## Results

### Clinical characteristics of the study participants

**Fig. 1:**
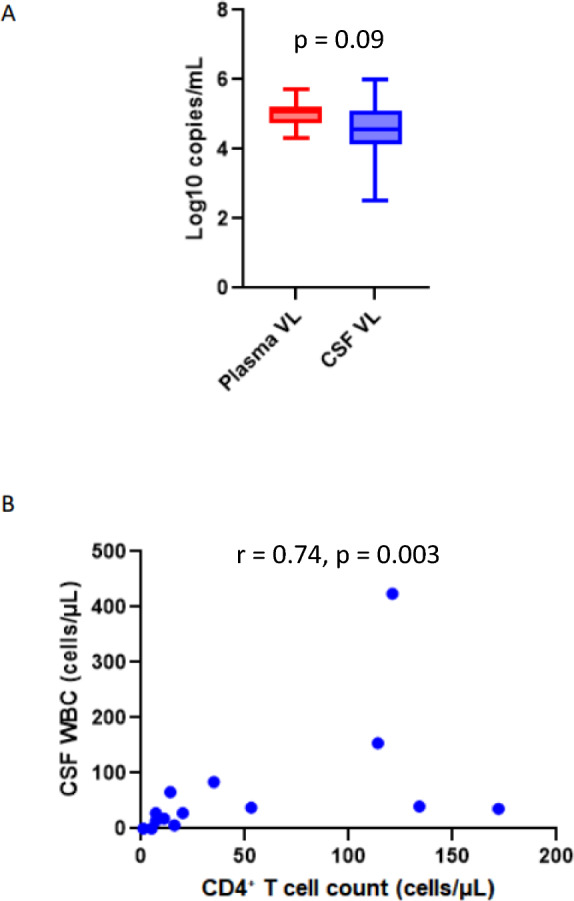
**(A):** Comparison of plasma and the CSF viral load**; (B)** Correlation between CD4 + T cell count and CSF total white blood cell count (WBC), correlation was assessed using Spearman’s correlation test

First, we assessed the clinical characteristics of the participants. We compared the median viral load between plasma and CSF compartments; the median viral load was slightly higher in plasma (5.10 log_10_ copies/mL) compared to the CSF compartment (4.55 log_10_ copies/mL) (p = 0.09) (Table 1; Fig. 1A). The median CD4 + T-cell peripheral blood count was 27.50 cells/μL, suggesting that the patients had advanced disease (Table 1). We additionally recorded the presence of white blood cells (WBCs) in the CNS (pleocytosis), a known indicator of disrupted blood–brain barrier (BBB) integrity and function [78]. Eleven of the twelve participants (92%) had > 5 WBCs/μL in the CSF, suggesting the occurrence of pleocytosis or disruption of BBB integrity (Table 1). Although, BBB disruption cannot be ruled out for the remaining participant, despite lower WBC counts, due to the possible occurrence of advanced CD4 + lymphopenia. A positive correlation was observed between the peripheral blood CD4 + T-cell count and the number of WBCs in the CSF (r = 0.74, p = 0.003) (Fig. 1B).

### Phylogenetic analysis and pseudovirus generation

To assess the inter- and intra-participant phylogenetic relatedness of the samples, we analysed the HIV-1C *env* sequences produced from the plasma and CSF compartments of the 12 participants (Appendix A1, Supp. Figure 1). In total, 45 HIV-1 *env* sequences were analysed across twelve participants. We also analysed 19 matched plasma and 21 CSF-derived sequences for seven participants as shown in Table 2. Approximately, 1–7 plasma sequences (median = 2), and 1–13 CSF sequences (median = 1) were selected in each participant based on their level of genetic diversity. Notably, sequence intermixing between the plasma and the CSF was observed in two participants (CM019 and CM098). In contrast, sequences from each compartment clustered separately in one participant (CM108). While for the remaining four participants, our ability to assess the extent of genotypic intermixing was limited by the small number of available sequences. We also analysed the genotypic relatedness of the unmatched plasma-derived sequences for two participants (CM054 and CM070), and the unmatched CSF-derived sequences for three participants (CM021, CM029, and CM094). We did not observe inter-participant phylogenetic intermixing of the sequences.

**Table 2:** The coreceptor usage of HIV-1 subtype C Envs U87-CD4 cells. Subtype B viruses were used as positive controls (YU2, NL4.3, 89.6)

PID	Sample type	HIV-1 Env clones	Coreceptor usage
**YU2**	Control	-	R5
**NL4.3**	Control	-	X4
**89.6**	Control	-	R3R5X4
**CM019**	CSF	CM019C.B	X4
	Plasma	CM019P.3	X4
		CM019P.B.3	R3X4
		CM019P.B.4	X4
		CM019P.B.5	X4
		CM019P.B.6	X4
		CM019P.H.1	R5X4
		CM019P.I.2	R5
**CM021**	CSF	CM021C.7.3	R5
**CM029**	CSF	CM029C.D.2	R5
**CM032**	CSF	CM032C.6.4	R5
	Plasma	CM032P.6.3	R5
**CM052**	CSF	CM052C.1	R5
	Plasma	CM052P.2.1	R5
		CM052P.2.2	R5
		CM052P.4.3	R5
**CM054**	Plasma	CM054P.11.6	R5
**CM070**	Plasma	CM070P.1	X4
**CM089**	CSF	CM089C.SGA_13	R5X4
		CM089C_SGA16	R3R5X4
	Plasma	CM089P_SGA.06	R5
**CM094**	CSF	CM094C.14.2	R5
	CSF	CM094C.6.2	R3R5
**CM098**	CSF	CM098C.C.3	R5
		CM098C.F.1	R5
		CM098C.F.3	R5
		CM098C.F.5	R5
		CM098C.G.1	R5
		CM098C.G.2	R5
		CM098C.G.4	R5
		CM098C.G.5	R5
		CM098C.H.1	R5
		CM098C.H.5	R5
		CM098C.I.1	R5X4
		CM098C.I.3	R5
		CM098C.J.4	R3R5X4
	Plasma	CM098P.B.1	R5X4
		CM098P.G.1	R5X4
**CM108**	CSF	CM108C.B.3	R3R5X4
		CM108C.D.8	R3R5
	Plasma	CM108P.1	R5X4
		CM108P.4	R3R5
		CM108P.D.5	R5X4
**CM132**	CSF	CM132C.1	R5X4
	Plasma	CM132P_SGA02	R5X4

### In vitro coreceptor usage of HIV-1C in U87-CD4 + cells

**Fig. 2:**
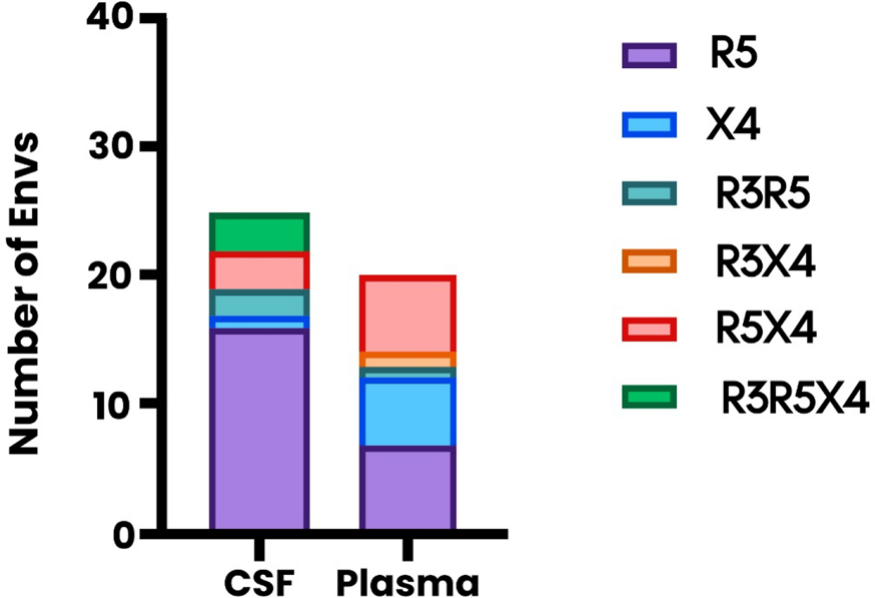
The coreceptor usage phenotypes in the CSF and the plasma across all participants. The proportions are based on the number of Envs that display each respective phenotype based on the overall coreceptor usage in Table 2. A total of 25 CSF and 20 plasma Envs were included in this analysis. Colour key: Purple – R5 Envs, Blue – X4 Envs, Green – R3R5 Envs, Orange – R3X4 Envs, Red – R5X4 Envs and Dark purple – R3R5X4 Envs

We sought to assess whether the phylogenetic clustering of the *env* variants were connected to their in vitro coreceptor usage in the different anatomical compartments. Therefore, we determined the in vitro coreceptor usage of plasma and CSF-derived clones using U87-CD4 + cells expressing CCR3, CCR5, or CXCR4 (Appendix A2, Supp. Figure 2; Table 2). For inter-participant comparison between the compartments, 16 out of 25 (64%) CSF-derived clones were R5, and only one (4%) clone was X4 (Table 2; Fig. 2). The remaining eight clones were either R5X4 or R3R5 (32%) (Table 2; Appendix A2, Supp. Figure 2: A-C). In contrast to the CSF-derived clones, only 7 (35%) plasma-derived clones were R5, 5 (25%) clones were X4, 6 (30%) clones were R5X4, and the remaining two clones (10%) were either R3R5, or R3X4 (Table 2; Fig. 2). These findings suggest that CSF-derived clones are mainly R5, whereas the presence of R5, X4, and R5X4 variants was comparable in plasma. Furthermore, CCR3-usage was observed in plasma and CSF compartments, but only in combination with CCR5 and/or CXCR4 usage. Plasma-derived clones had a wider variation of in vitro coreceptor usage, and CXCR4 usage was more frequent in plasma compared to the CSF. Additionally, of the seven clones capable of using CCR3, five were observed within the CSF, and only two were observed in the plasma compartment.

For intra-participant comparison (seven participants) between the compartments, concordant or overlapping coreceptor usage was observed in four participants (57%) (CM019, CM032, CM052 and CM132). Only one participant (14%, CM089), displayed discordant coreceptor usage between the CSF and plasma which also corresponded with distinct clustering of phylogenetic sequences between the two compartments. Notably, for CM019, intermixing of sequences was observed between the two compartment-derived sequences, compared to CM052, where the compartment-derived phylogenetic sequences clustered separately. Additionally, one participant (14%, CM108) which also displayed distinct clustering of the phylogenetic sequences between compartments, showed mostly discordant coreceptor usage between the two compartments, where only one CSF clone and one plasma clone (CM108C.D.5 and CM108P.4) shared the same coreceptor usage phenotype (R3R5). In contrast, another participant (14%, CM098) which displayed phylogenetic intermixing of sequences between the compartments, mostly had distinct and discordant coreceptor usage between the compartments, with only one viral clone from thirteen, showing the same coreceptor phenotype as the plasma. Due to the small number of sequences analysed, characterisation of phylogenetic intermixing was limited to participants who had more than three sequences available. Therefore, intraparticipant comparison of in vitro coreceptor usage showed overlapping viral phenotypes between plasma- and CSF- derived Env variants in most of the participants, with some evidence of phenotypic viral compartmentalisation. Discordant coreceptor usage between the compartments did not consistently correspond with phylogenetic clustering between the compartments. Furthermore, monotropic (R5 and X4), dual-tropic (R5X4, R3R5, and R3X4) and tri-tropic (R3R5X4) Env variants were detected in both the plasma and CSF of this participant population.

### Genotypic characteristics linked to the coreceptor usage

**Fig. 3:**
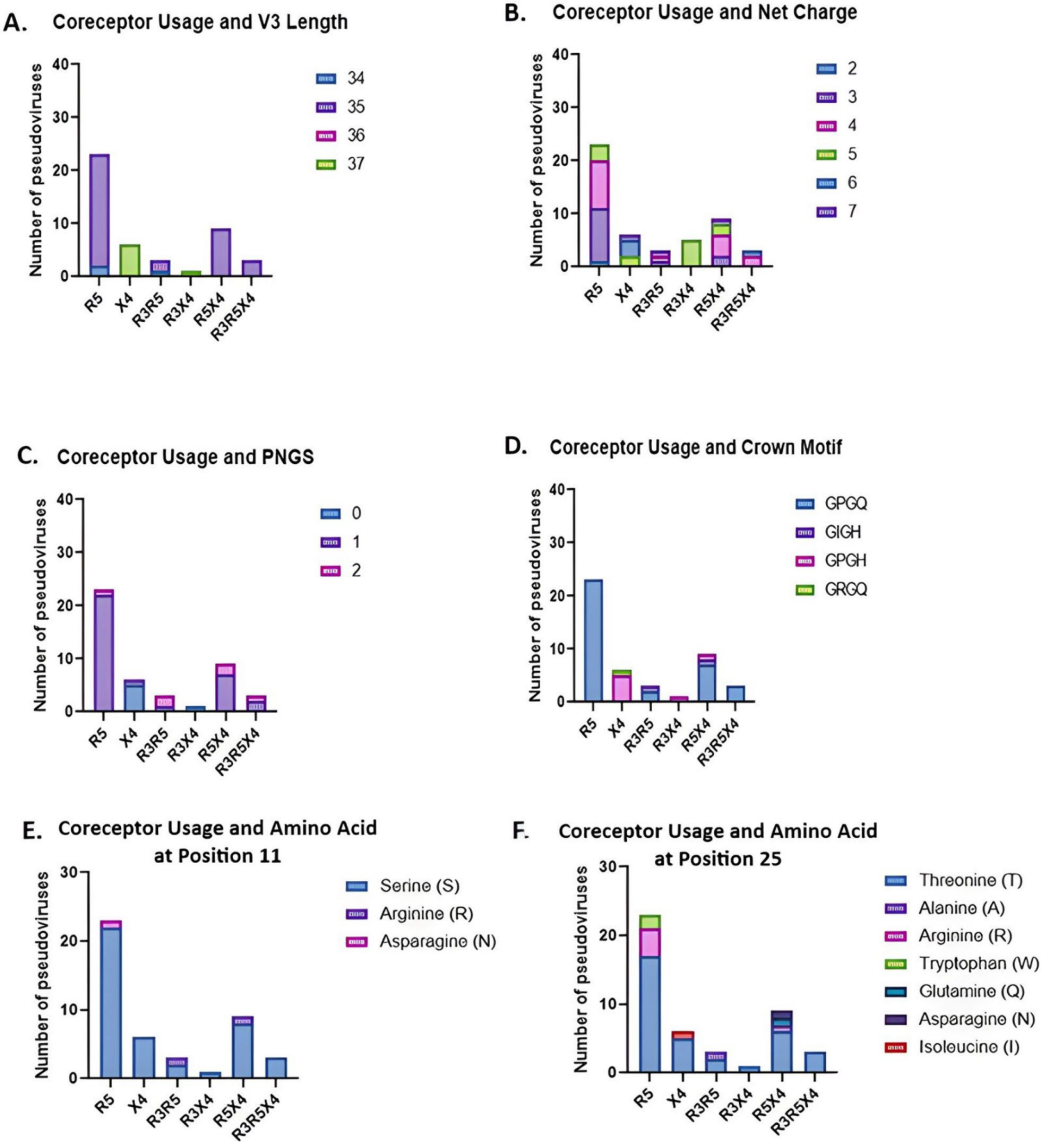
HIV-1C Env V3 properties. The overall coreceptor usage of the Env and other properties including **(a)** the V3 length; **(b)** net charge; **(c)** number of potential N-linked glycosylation sites (PNGS); **(d)** crown motif; **(e & f)** amino acids at positions 11 and 25 are indicated

Since we observed a variety of coreceptor usage profiles across the participants, we next evaluated whether there were any genotypic characteristics linked to the coreceptor usage profiles we observed. Briefly, we analysed HIV-1C Envs genotypic properties associated with in vitro coreceptor usage through amino acid (AA) signatures linked to CCR5 and CXCR4 usage [28]. Using a sequence alignment of the HIV-1C Env clones and in vitro coreceptor data, we assessed characteristics including the length, net charge, number of potential N-linked glycosylation sites (PNGS), substitutions in the crown motif, and residues at positions 11 and 25 (Fig. 3A-F). The majority of X4 clones had longer V3 loops (37 AAs), higher V3 net charges (> 5 +) and lacked PNGS compared to variants that were classified as R5, R5X4, R3R5 and R3R5X4 clones (Fig. 3A-C). Most X4 and all R3X4 clones exhibited the GPGH or GRGQ motifs. In contrast, R5 clones had a conserved GPGQ motif. CCR5-using variants with expanded coreceptor usage (R3R5, R5X4, R3R5X4), mostly exhibited GPGQ motifs, with a limited occurrence of GIGH and GPGH (Fig. 3D). Regarding amino acid positions, both R5 and X4 clones had a conserved serine at position 11 however, one (4%) R5 clone (CM098.C.F.5) exhibited asparagine (N) at position 11. Arginine (R) residues at this position was observed in R3R5, and R5R4 plasma clones in one of the participants (CM108) (Fig. 3E, Appendix A3, Supp. Figure 3). While CSF clones did not have arginine residues in this position. Similarly, at position 25, while most clones had a conserved threonine (T), 4 (17%) R5 clones exhibited arginine (R) and two (9%) exhibited tryptophan (W) (Fig. 3F, Appendix A3, Supp. Figure 3). In contrast, one X4 clone had isoleucine (I) residues, while R5X4 clones showed a wider range of residues in this position (Fig. 3F). Our findings suggest that the longer V3 loops, lack of PNGS, higher net charge and the presence of GPGH or GRGQ motifs could be associated with X4 and R3X4 viruses. Whereas the shorter V3 loops, presence of PNGS, lower net charge and presence of GPGQ motifs may characterise R5 variants.

## Discussion

This study investigated the in vitro coreceptor usage and V3 loop characteristics associated with CCR5 and CXCR4 usage phenotypes in plasma and cerebrospinal fluid (CSF) of HIV-1C and cryptococcal meningitis co-infected patients. Our findings revealed that the CNS environment appears to limit HIV-1C mainly to CCR5 usage, despite a broader coreceptor usage profile observed within the plasma, including CCR5, CXCR4, and combinations of CCR5/CXCR4/CCR3 usage. In contrast, CXCR4 usage, while present in the CSF, was more frequent in the plasma than in the CSF. Similarly, previous studies have also reported high CCR5 usage in CSF-derived clones compared to plasma-derived clones for advanced and chronic HIV-1 infection across different HIV subtypes (AE, B, C, D) [24, 49, 51].

CCR3-usage was observed in both compartments, but notably only when accompanied by CCR5 and/or CXCR4 usage. Furthermore, data from one study [51] showed that while CCR3 usage was more frequent in the CSF compared to the plasma compartment, CCR3 usage was not observed in isolation from other coreceptors. This therefore corroborates our results, suggesting that HIV-1 circulating in the CNS, while capable of using CCR3, may be dependent on another receptor such as CCR5 for entry. Therefore, it is unclear whether CCR3 usage is required or obligatory for the persistence of these variants within the CSF. Notably, others reported a higher frequency of CCR3/CCR5 usage in the CSF compared to our study, which may be due either to our small sample size or to differences in HIV subtypes analysed (HIV-1B, AE, C and D participants) [51], as we only analysed HIV-1C participants. In comparison to the varied stages of infection (CD4 count ranged from 27- 3820 cells/uL) and the presence of various AIDS-defining diseases including HIV-associated dementia (HAD) in that study [51], participants in this study were all in the advanced stage of disease and co-infected only with cryptococcal meningitis. Together, these studies suggest that most CSF-derived clones use CCR5 exclusively or in combination with CCR3 and/or CXCR4. This highlights the crucial role of CCR5 for viral entry, even in advanced infection. The crucial role of CCR5 in advanced infection is further evidenced by the fact that in other studies and ours, some chronically infected individuals display no expanded coreceptor usage profile, and only harbour R5 variants [79]. The presence of variants that use CCR3 or CXCR4 may represent viral evolution in response to changes in target cell preference or prevalence, or the immunological microenvironment. Our findings also highlight that HIV-1C predominantly uses CCR5, with frequent usage of CCR3 and CXCR4 within the plasma compartment during the advanced stage of HIV-1 subtype C infection with cryptococcal meningitis coinfection.

Previous studies indicate that people living with HIV can show either equilibration or compartmentalization of viral genetic variants between peripheral blood and the CNS [80–83]. Pleocytosis has been associated with equilibration of genetic variants between peripheral blood and the CNS due to viral trafficking as a result of breakdown of the blood–brain barrier [78, 80]. Most participants in our study had pleocytosis and yet we were still able to detect genetic and phenotypic compartmentalization between the blood and CNS compartments even with bulk sampling. Although we are limited by the cross-sectional sampling in our study, we speculate that pleocytosis in these study participants was not enough to fully equilibrate the compartmentalized populations that may have existed in the two distinct anatomical regions prior to cryptococcocal meningitis.

Finally, we confirmed that specific V3 loop genetic signatures are associated with either CCR5- or CXCR4 usage. A longer V3 loop length, higher V3 net charge, lack of N-linked glycosylation sites and the GPGH or GRGQ motifs were associated with CXCR4 usage. While the shorter V3 loop length, lower net charge, the presence of one or two PNGS and the GPGQ motif were associated with CCR5 usage.

Taken together, our findings indicate variations in coreceptor usage between plasma and CSF compartments of some participants during the advanced stage of HIV-1C and CM coinfection. The prevalence of CXCR4-using viruses is relatively high during chronic infection than early infection and may have been underestimated previously, with initial evidence suggesting that CXCR4 usage was rare or uncommon [36, 84–87]. This indicates potential limitations in using entry inhibitors that only block CCR5 usage (e.g., MVC). We further confirmed that longer V3 loop (37 amino acids), lack of N-linked glycans and increased net charge could be predictors for CXCR4 usage. We also identified amino acid signatures that may differentiate between CCR5 and CXCR4 variants. Our study therefore highlights that coreceptor usage profiles in advanced HIV-1C infection can vary between the plasma and the CSF within the same individual. This therefore underscores why detailed characterisation of compartment-specific viral populations is essential for understanding viral pathogenesis and for guiding appropriate use of entry inhibitors, particularly those targeting CCR5 such as MVC, which may not be effective when CXCR4-using or multi-tropic variants are present.

This study had several limitations, including a small sample size, as we could not generate and compare multiple pseudoviruses per compartment for every participant. Additionally, by using bulk PCR to generate some sequences, the possibility of missing minority variants is high, as the technique tends to select for dominant variants in the sample. Another significant limitation is the absence of a control group of HIV-1C mono-infected participants, as only samples from HIV-1 and CM co-infected participants were available. Future research may include a control group (e.g., recently infected participants, participants with HIV-associated neurocognitive disorder, or HIV-TB co-infected patients) to better understand the coreceptor usage profiles specific to patients with advanced HIV-1C disease. Additionally, we did not assess coreceptor usage using primary cells, or other physiologically relevant T cells or monocytes/macrophages cell lines. Though several regions of Env may influence coreceptor usage [88–93], we focused only on characterising the V3 region as it is the main determinant of coreceptor usage. Hence, future research should assess other regions of the HIV-1 Env, including synonymous and non-synonymous nucleotide substitutions to the gp120 and gp41 regions.

## Conclusion

This study assessed the in vitro coreceptor usage in plasma and CSF-HIV-1C-derived clones in ART-naïve participants co-infected with CM. We demonstrated that coreceptor usage of HIV-1C is heterogeneous in plasma and CNS compartments, whereby monotropic, dual-tropic or tri-tropic variants can circulate among other Env phenotypes within a compartment, and differences in coreceptor usage can be detected across compartments. Additionally, further insights into the biological context of HIV-1C usage of CXCR4 and CCR3 was demonstrated. Overall, our study has contributed to the understanding of HIV-1C pathogenesis in advanced disease and across the blood and CNS compartments and may be used to inform the design of treatments targeting the entry step of the HIV-1 life cycle.

## Supplementary Information

Supplementary Material 1.

Supplementary Material 2.

## Data Availability

The data generates and/or analysed in this study, including the HIV-1C Env HIV-1C sequences are available in GenBank and formed part of a larger sequence set with accession numbers: MF284812 – MF284921 and MF284922 – MF285062.

## Abbreviations

HIV-1C: HIV-1 Subtype C
CNS: Central nervous system
Env: Envelope protein (of HIV-1)
CSF: Cerebrospinal fluid
PLWH: People living with HIV
ART: Antiretroviral therapy
PrEP: Pre-Exposure prophylaxis
STIs: Sexually transmitted infections
MVC: Maraviroc (CCR5 antagonist)
R5: CCR5-using viruses
X4: CXCR4-using viruses
R5X4: Dual-tropic viruses using CCR5 and CXCR4
CM: Cryptococcal meningitis
cART: Combined antiretroviral therapy
C-IRIS: Cryptococcosis-associated Immune Reconstitution Inflammatory Syndrome
WBC: White blood cell
BBB: Blood–Brain Barrier
CrAg: Cryptococcal antigen
RNA: Ribonucleic acid
cDNA: Complementary DNA
SGA: Single genome amplification
PCR: Polymerase chain reaction
kb: Kilobase
DNA: Deoxyribonucleic acid
ATCC: American type culture collection
NIH: National institutes of health
AIDS: Acquired immunodeficiency syndrome
DMEM: Dulbecco’s modified eagle medium
PBS: Phosphate-Buffered saline
RLU: Relative light unit
AA: Amino acid
PNGS: Potential N-linked glycosylation site
HAD: HIV-Associated dementia
TB: Tuberculosis
BREC: Biomedical research and ethics committee (UKZN)
PRF: Poliomyelitis research foundation
EDCTP: European & developing countries clinical trials partnership
IAVI: International AIDS vaccine initiative
NIAID: National Institute of Allergy and Infectious Diseases
CFAR: Center for AIDS research
NCI: National cancer institute
NICHD: National institute of child health and human development
NHLBI: National heart, lung, and blood institute
NIDA: National institute on drug abuse
NIMH: National institute of mental health
NIA: National institute on aging
FIC: Fogarty international center
OAR: Office of AIDS research
SANTHE: Sub-Saharan african network for TB/HIV research excellence
DELTASL: Developing excellence in leadership, training and science in africa
