# Supplementary material for: Coreceptor usage of plasma and cerebrospinal fluid-derived HIV-1 subtype C variants
Source: Virol J. 2026 Apr 4;23:125. doi: 10.1186/s12985-026-03150-0 (PMC13192158; doi:10.1186/s12985-026-03150-0)
Supplement: Supplementary file 2 — Supplementary Material 2. [file 12985_2026_3150_MOESM2_ESM.pptx]

## Slide 1
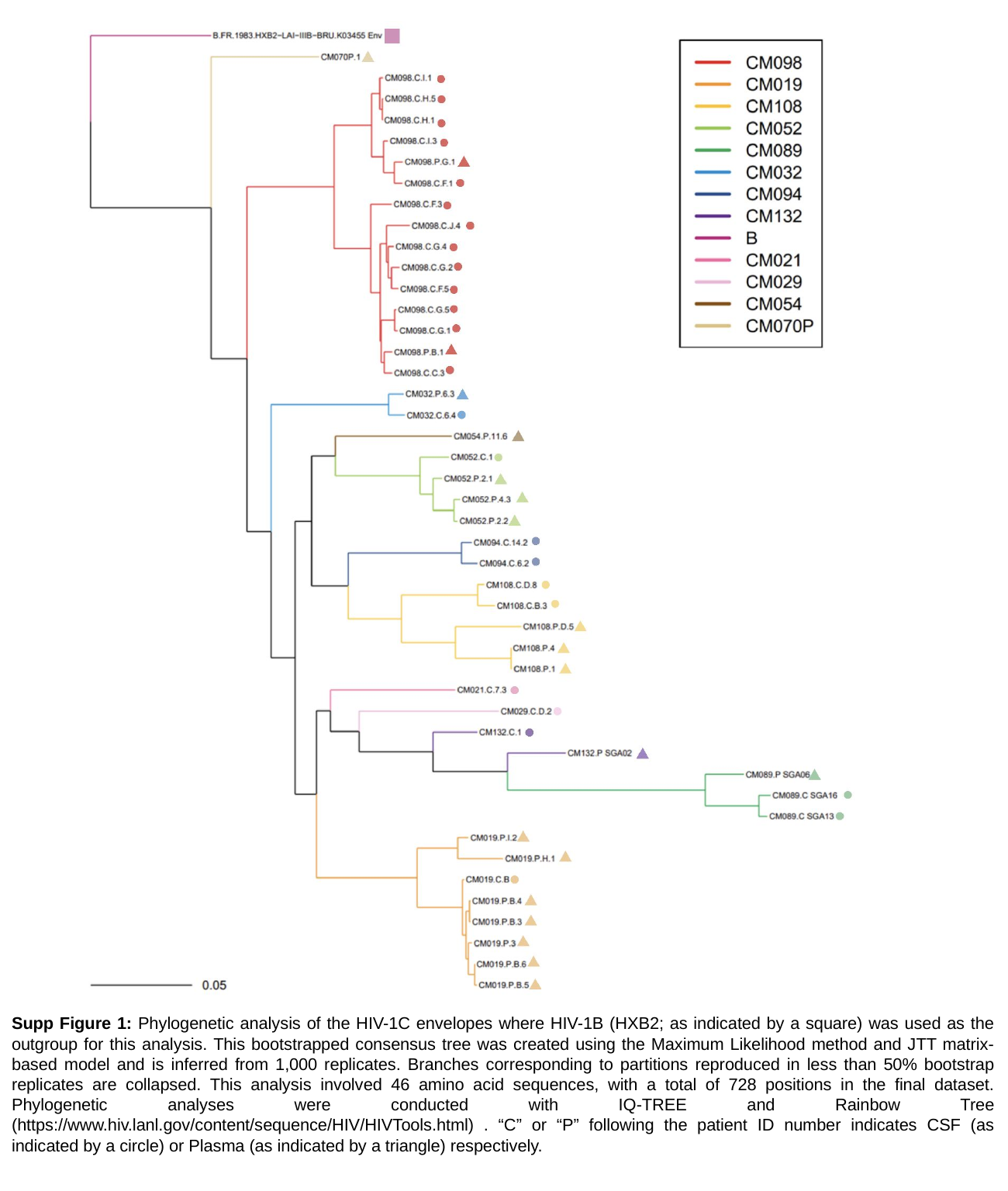

Supp Figure 1: Phylogenetic analysis of the HIV-1C envelopes where HIV-1B (HXB2; as indicated by a square) was used as the outgroup for this analysis. This bootstrapped consensus tree was created using the Maximum Likelihood method and JTT matrix-based model and is inferred from 1,000 replicates. Branches corresponding to partitions reproduced in less than 50% bootstrap replicates are collapsed. This analysis involved 46 amino acid sequences, with a total of 728 positions in the final dataset. Phylogenetic analyses were conducted with IQ-TREE and Rainbow Tree (https://www.hiv.lanl.gov/content/sequence/HIV/HIVTools.html) . “C” or “P” following the patient ID number indicates CSF (as indicated by a circle) or Plasma (as indicated by a triangle) respectively.

## Slide 2
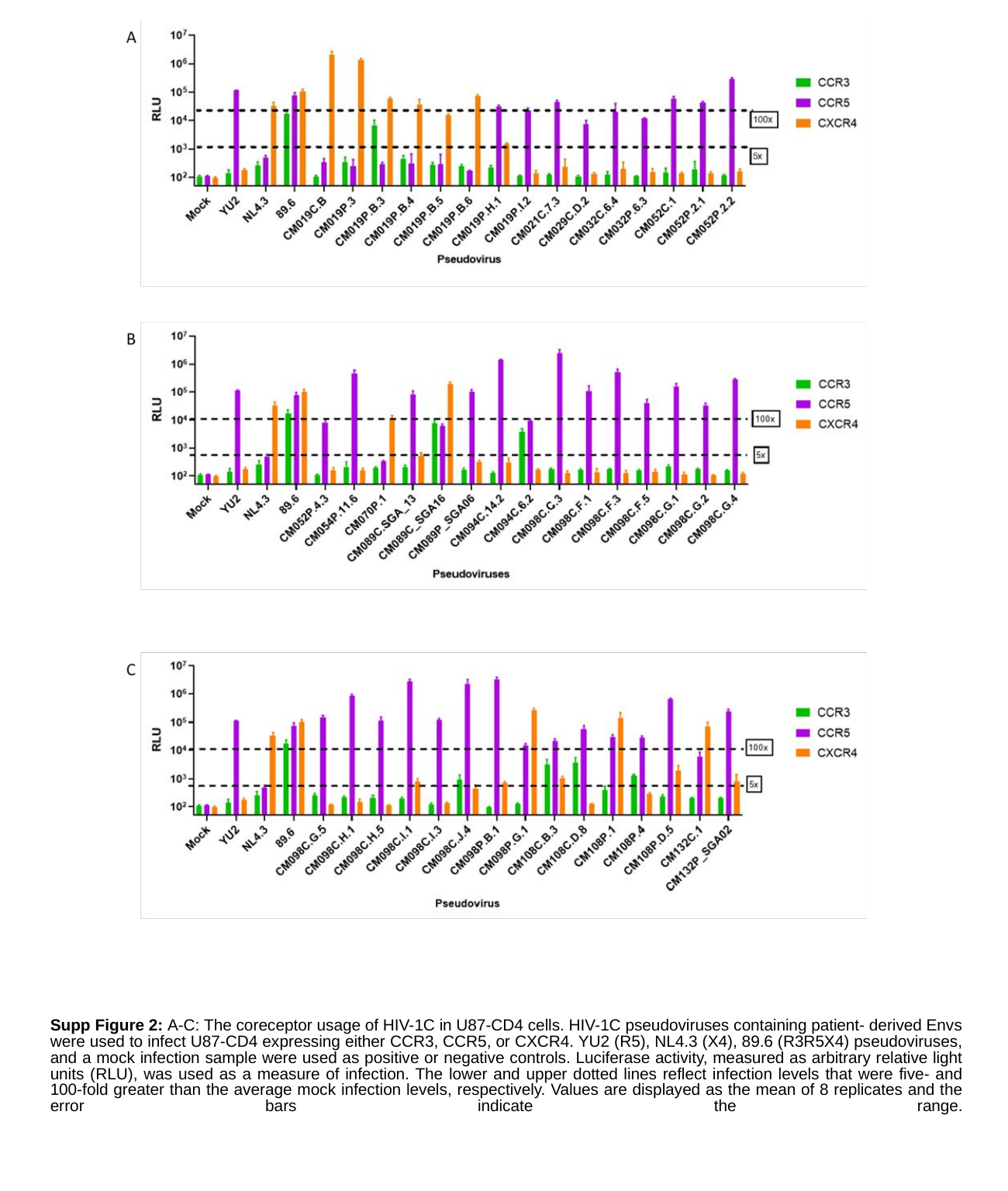

# Supp Figure 2: A-C: The coreceptor usage of HIV-1C in U87-CD4 cells. HIV-1C pseudoviruses containing patient- derived Envs were used to infect U87-CD4 expressing either CCR3, CCR5, or CXCR4. YU2 (R5), NL4.3 (X4), 89.6 (R3R5X4) pseudoviruses, and a mock infection sample were used as positive or negative controls. Luciferase activity, measured as arbitrary relative light units (RLU), was used as a measure of infection. The lower and upper dotted lines reflect infection levels that were five- and 100-fold greater than the average mock infection levels, respectively. Values are displayed as the mean of 8 replicates and the error bars indicate the range.

## Slide 3
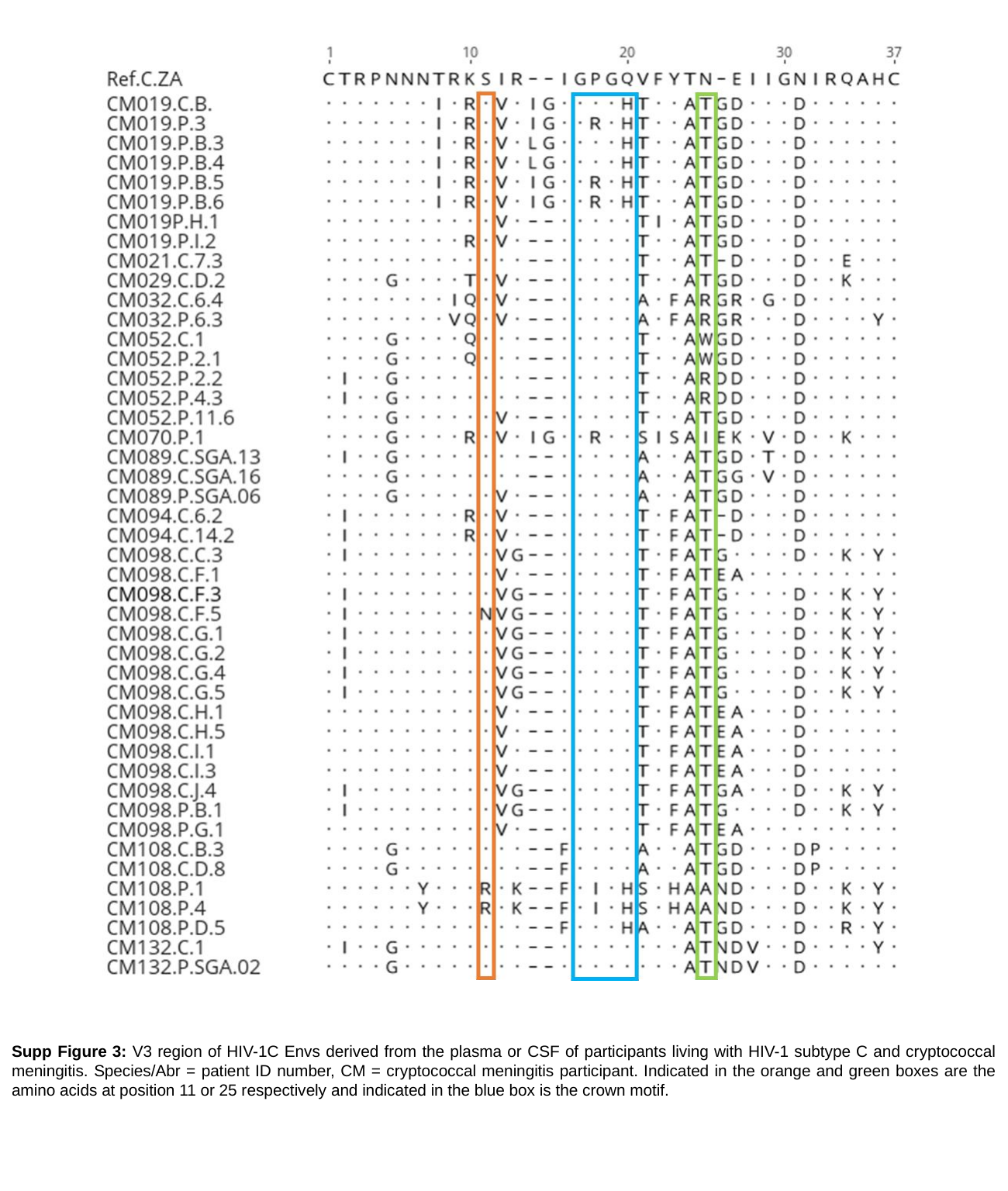

Supp Figure 3: V3 region of HIV-1C Envs derived from the plasma or CSF of participants living with HIV-1 subtype C and cryptococcal meningitis. Species/Abr = patient ID number, CM = cryptococcal meningitis participant. Indicated in the orange and green boxes are the amino acids at position 11 or 25 respectively and indicated in the blue box is the crown motif.
